# The Effects of Telemonitoring on Patient Compliance With Self-Management Recommendations and Outcomes of the Innovative Telemonitoring Enhanced Care Program for Chronic Heart Failure: Randomized Controlled Trial

**DOI:** 10.2196/17559

**Published:** 2020-07-08

**Authors:** Hang Ding, Rajiv Jayasena, Sheau Huey Chen, Andrew Maiorana, Alison Dowling, Jamie Layland, Norm Good, Mohanraj Karunanithi, Iain Edwards

**Affiliations:** 1 RECOVER Injury Research Centre Faculty of Health and Behavioural Sciences The University of Queensland Brisbane Australia; 2 The Australian e-Health Research Centre Commonwealth Scientific & Industrial Research Organisation Health & Biosecurity Brisbane Australia; 3 Faculty of Medicine The University of Queensland Brisbane Australia; 4 The Australian e-Health Research Centre Commonwealth Scientific & Industrial Research Organisation Health & Biosecurity Melbourne Australia; 5 School of Physiotherapy and Exercise Science Curtin University Perth Australia; 6 School of Physiotherapy and Exercise Science Australia Faculty of Health Sciences Curtin University Perth Australia; 7 Allied Health Department and Advanced Heart Failure and Cardiac Transplant Service Fiona Stanley Hospital Perth Australia; 8 Department of Community Health Peninsula Health Melbourne Australia; 9 Department of Cardiology Peninsula Health Melbourne Australia

**Keywords:** heart failure, digital health, telemonitoring, remote monitoring, patient compliance, randomized controlled trial

## Abstract

**Background:**

Telemonitoring enables care providers to remotely support outpatients in self-managing chronic heart failure (CHF), but the objective assessment of patient compliance with self-management recommendations has seldom been studied.

**Objective:**

This study aimed to evaluate patient compliance with self-management recommendations of an innovative telemonitoring enhanced care program for CHF (ITEC-CHF).

**Methods:**

We conducted a multicenter randomized controlled trial with a 6-month follow-up. The ITEC-CHF program comprised the provision of Bluetooth-enabled scales linked to a call center and nurse care services to assist participants with weight monitoring compliance. Compliance was defined a priori as weighing at least 4 days per week, analyzed objectively from weight recordings on the scales. The intention-to-treat principle was used to perform the analysis.

**Results:**

A total of 184 participants (141/184, 76.6% male), with a mean age of 70.1 (SD 12.3) years, were randomized to receive either ITEC-CHF (n=91) or usual care (control; n=93), of which 67 ITEC-CHF and 81 control participants completed the intervention. For the compliance criterion of weighing at least 4 days per week, the proportion of compliant participants in the ITEC-CHF group was not significantly higher than that in the control group (ITEC-CHF: 67/91, 74% vs control: 56/91, 60%; *P*=.06). However, the proportion of ITEC-CHF participants achieving the stricter compliance standard of at least 6 days a week was significantly higher than that in the control group (ITEC-CHF: 41/91, 45% vs control: 23/93, 25%; *P*=.005).

**Conclusions:**

ITEC-CHF improved participant compliance with weight monitoring, although the withdrawal rate was high. Telemonitoring is a promising method for supporting both patients and clinicians in the management of CHF. However, further refinements are required to optimize this model of care.

**Trial Registration:**

Australian New Zealand Clinical Trial Registry ACTRN12614000916640; https://www.anzctr.org.au/Trial/Registration/TrialReview.aspx?id=366691

## Introduction

Chronic heart failure (CHF) is a severe chronic disease that affects more than 26 million people worldwide [1]. It significantly reduces the health-related quality of life and increases the risk of hospitalization and mortality [1]. To improve health outcomes, it is recommended that patients with CHF undertake self-management, such as daily monitoring of body weight to assess fluid balance and seek early clinical support in the event of symptoms, which may indicate decompensation. This has been consistently outlined by evidence-based clinical guidelines for CHF [2,3] and is practically supported by CHF clinics and rehabilitation programs in standard care. Despite these clinical efforts, patient compliance with self-management recommendations is often suboptimal for activities such as body weight recording, fluid restrictions, and medication adherence [4]. Time constraints [5], limited knowledge [6], and insufficient ongoing clinical support [7] are some of the reported barriers to the self-management of CHF. Poor compliance with self-management recommendations often leads to delays in essential treatment [4] and increases the risk of mortality and hospitalization [8].

In recent years, there has been significant research interest in telemonitoring as an innovative approach to remotely assist patients with CHF in self-managing their health [9]. However, to date, only 2 studies, to our knowledge, have evaluated patient compliance with weight monitoring in a randomized controlled trial (RCT) [10,11]. Although they demonstrated a higher rate of *compliant* participants in the telemonitoring arm (telemonitoring vs usual care: 88.6% vs 70.9% [10] and 91.7% vs 67.4% [11]), the studies relied on self-report, which is known to be influenced by recall bias [12]. In addition, the definition of *compliance* was loosely defined based on terms such as *most of the time* or *all of the time* and, hence, was not sufficiently accurate to reflect the daily weight monitoring recommendation. Moreover, patient adherence to telemonitoring systems has often been found to be low, even in large, well-designed RCTs (55% [13] and 55.4% [14]). This has led to an ongoing debate about the practicality of using telemonitoring to improve CHF care [13-15]. Therefore, further rigorous research for evaluating patient compliance is needed in telemonitoring studies for CHF care.

We evaluated an innovative telemonitoring enhanced care program for CHF (ITEC-CHF) in an open multicenter RCT. The ITEC-CHF program focused on assisting patients in daily weight monitoring and engaging with nurse-supported care in the event of weight fluctuations. This study aimed to examine whether the ITEC-CHF program improved patient compliance with weight monitoring as well as other self-management behaviors and health outcomes.

## Methods

### Study Design

The protocol for the ITEC-CHF study has been previously published [15]. Images of the user interface and the Bluetooth-enabled scales are provided in Multimedia Appendices 1 and 2. In this study, patients with CHF were recruited from 2 trial sites in Australia: one in Victoria (VIC) and one in Western Australia (WA). The trial sites were at 2 hospitals in VIC and WA, respectively. This study complies with the Declaration of Helsinki. All participants provided written informed consent. The clinical trial protocol was approved by the Human Research Ethics Committee at Peninsula Health, VIC (HREC reference: HREC/14/PH/27), and Royal Perth Hospital, WA (reference: 15-081 and reference: HR 181/2014), Australia. Participants were enrolled from January 2015 to October 2017. The latest data collection of hospitalizations and emergency department (ED) presentations was conducted in September 2018.

### Randomization and Masking

Participants in the trial were individually randomized with an allocation ratio of 1:1 to receive either ITEC-CHF or usual care (control) for 6 months. Randomization was stratified by the 2 trial sites (VIC and WA) to ensure that the allocation ratio was consistent at each site. A block method was used to achieve a balanced number of participants between the ITEC-CHF and control groups throughout the trial. The random allocation assignments were sealed in opaque envelopes. Data analysts generated the randomization sequence and were blinded to the trial because of the use of deidentified patient data.

### Inclusion and Exclusion Criteria

The inclusion criteria were as follows: patients (1) with CHF with reduced ejection fraction (EF; ie, EF≤40%), (2) able to weigh themselves safely, (3) aged at least 18 years, (4) having a regular personal general practitioner (GP) or agreeing to use a designated GP, (5) with a permanent residential address, and (6) without significant cognitive impairments. The exclusion criteria were as follows: (1) patients with expected survival <12 months, (2) patients with end-stage renal failure on dialysis, (3) long-term nursing home residents, or (4) patients participating in any other clinical trial.

### Interventions

At baseline, control participants were provided with a standard package of a paper-based diary and the *Living Well with Chronic Heart Failure* booklet, produced by the Heart Foundation of Australia. They were instructed to maintain their usual CHF care, as provided by clinics specialized in CHF and primary care physicians, and to undertake CHF self-management as previously instructed. Each participant was also provided with an electronic weight scale (FORA TN'G W550; ForaCare) and asked to use the scale to measure their body weight daily, immediately after waking, following voiding, without shoes, in light clothing, and before taking medication. Approximately every 3 months, project nurses visited the participants to download the weight entries from the weight scale.

Participants in the ITEC-CHF group received the same resources as those in the control group, in addition to the ITEC-CHF program. The ITEC-CHF consists of 3 major components: remote body weight monitoring, structured telephone support, and nurse-led collaborative care. The service was integrated with a telephone call center (MEPACS, VIC, Australia) and community nurse care services at the trial sites in VIC and WA. Each participant was provided with an electronic weight scale and a computer tablet (Galaxy Tab A, Samsung). Participants were asked to weigh themselves using the procedure described for the control group. After the measurement, the weight entry was automatically transmitted from the weight scale to the tablet via a wireless Bluetooth function. The tablet was preloaded with an Android app (Medtech Global). This app received the weight entry and uploaded the entry to a software package called Manage My Health (MMH; Medtech Global). A rule-based decision support system (a web app) in MMH automatically monitored the uploaded weight entries in real time. In response to the weight data generated by telemonitoring, 6 types of alerts were possible: (1) *rapid weight fluctuation* (an increase or decrease of 2 kg over 2 days), (2) *slow weight fluctuation* (an increase or decrease of 5 kg over 28 days), (3) *low-risk weight fluctuation* (an increase or decrease of 1 kg over 24 hours), (4) missed weight measurement, (5) low level of tablet battery, and (6) tablet connection lost. In the event of a *rapid weight fluctuation*, project nurses were alerted, and they called the participant to assist him or her in assessing symptoms and activating their CHF action plan, such as attending their GP, visiting a CHF clinic, or presenting to an ED as indicated. For an alert of *slow weight fluctuations*, the project nurses assisted the participants in assessing CHF symptoms and arranging clinical reviews at the participants’ GP or CHF clinics as indicated. For *low-risk fluctuations*, a questionnaire was automatically triggered and sent to the participant’s computer tablet to help him or her determine the need for further clinical follow-up. Finally, the generated alerts were distributed to project nurses and/or the MEPACS call center in the ITEC-CHF program. Call operators at the center responded to the alerts in real time (24 hours, 7 days a week), focusing on reminding participants to weigh themselves if they had not done so before 10 AM, helping assess CHF symptoms and manage diet, and arranging a nurse follow-up if needed. The project nurses reviewed their alert requests on weekdays and followed up with the participants via a telephone call. Some participants were unable to monitor their body weight for a short period, such as when they were hospitalized for medical treatment, traveled away from home, or experienced unresolved technical issues. Under such conditions, they were required to notify the call center, and weight monitoring was skipped. If a participant notified the call center to skip the monitoring for a period, the telemonitoring intervention was then *switched off* during the skipped period, and the call center did not receive any alerts from the participant and provide intervention until the skipped period ended. Monitoring days that were *skipped* were still included in the per-protocol analysis for the ITEC-CHF group (described in the Primary and Secondary Outcomes section).

### Primary and Secondary Outcomes

The primary outcome was in compliance with weight monitoring. The monitoring frequency was calculated as average weight monitoring days per week during the 6-month assessment period (monitoring frequency=weight monitoring days/180 days×7 days/week [for 6 months: 6 months×30 days/month=180 days]). A weight monitoring day was determined if at least one weight entry was practically recorded on the weight scale on that day, irrespective of time. In total, 2 frequencies were employed in the examination. One was that the participant monitored their weight on at least four days per week. This frequency reflects the compliance threshold of *most of the time*, as previously applied in questionnaire-based assessments [4,12]. The other frequency was at least 6 days per week, which more closely aligns with the advice for patients to monitor their weight *daily*.

Secondary outcomes included patient compliance with weight monitoring based on a per-protocol analysis (only undertaken in participants who completed the trial) and an analysis of other guideline recommendations assessed by the Heart Failure Compliance Questionnaire [12], health-related quality of life (five-dimension EuroQol, EQ-5D [16]), 6-min walk test distance [17], psychological state (cardiac depression scale short form 2 [18]), frailty (clinical frailty index [19]), and clinical outcomes of CHF-related and all-cause hospitalizations and ED presentations. CHF-related events were determined by using the International Classification of Diseases, Tenth Revision, Clinical Modification, diagnosis codes (Multimedia Appendix 3) [20]. Furthermore, we reported the alerts provided in ITEC-CHF and days when ITEC-CHF participants requested to *skip* weight monitoring.

### Statistical Analysis

In the evaluation, a chi-square test was applied to analyze continuous variables such as age, and a Fisher exact test was used to compare categorical variables such as sex and subgroups of participants under a given weight monitoring frequency (participants who achieved a given monitoring frequency vs participants who did not achieve the monitoring frequency). The Wilcoxon signed-rank test was used to compare compliance scores from questionnaire-based assessments. An analysis of covariance model [21] was used to evaluate the improvement or change in the outcome variables between the 2 groups with an adjustment for baseline. The Andersen-Gill model [22] with an adjustment for sex and age was used to analyze the hazard of hospitalizations and ED presentations. The 95% CI was estimated for the hazard function in each group. A *P* value<.05 was considered statistically significant. The analysis was conducted using RStudio version 1.1.383 (RStudio Inc) [23] with the R package of *survival* version 2.43-3. The intention-to-treat principle was applied to the analysis of the primary outcome of patient compliance with weight monitoring. It was also applied to the analysis of the hazards of hospitalization and ED. In the intention-to-treat analysis, all participants in the RCT were included. A per-protocol analysis was also applied to weight monitoring as a secondary outcome, only including participants who did not discontinue in the trial. A complete case analysis, which restricts the analysis to individuals with complete data, was used to analyze improvements in questionnaire-based assessments and 6-min walk distances.

## Results

**Figure 1:**
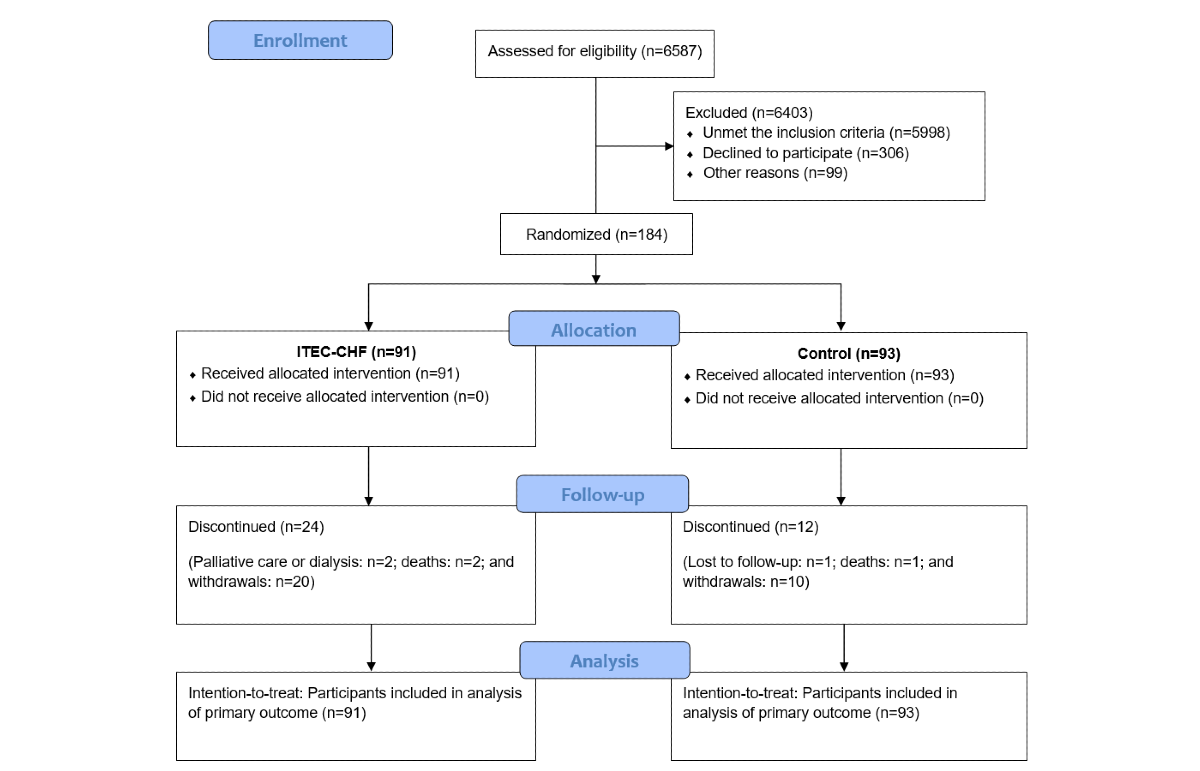
Patient enrollment and disposition. ITEC-CHF: innovative telemonitoring enhanced care program for chronic heart failure.

A total of 6587 patients were screened for eligibility. Among them, 6403 patients were excluded because of failure to meet the inclusion criteria (n=5998), declined (n=306), or for other reasons (n=99), such as losing contact with the patient (Figure 1). Finally, 184 patients were randomized to the ITEC-CHF (n=91) and control (n=93) groups. During the 6-month intervention period, 24 participants in the ITEC-CHF group discontinued (palliative care or dialysis: n=2; deaths: n=2; and withdrawals: n=20), and 12 participants in the control group discontinued (lost to follow-up: n=1; deaths: n=1; and withdrawals: n=11). According to the intention-to-treat principle, all randomized participants (ITEC-CHF: n=91; control: n=93) were included in the analysis of the primary outcomes of patient compliance with weighing. They were also included in the analysis of the hazards of hospitalization and/or ED presentation.

### Baseline Characteristics of the Participants

There were no significant differences between the characteristics of the ITEC-CHF and control groups at baseline (Table 1). The mean ages of the participants in the ITEC-CHF and control groups were 69.5 (SD 12.3) years and 70.8 (SD 12.4) years, respectively. Participants were predominantly male, and a high proportion of participants were diagnosed with type 2 diabetes, chronic obstructive pulmonary disease, or asthma. Common medications included angiotensin-converting enzyme inhibitors, beta-blockers, loop diuretics, and/or aldosterone receptor antagonists.

**Table 1:** Patient baseline characteristics.

Characteristic^a^		Victoria			Western Australia			Total	
		ITEC-CHF^b^ (n=42)	Control (n=42)	ITEC-CHF (n=49)		Control (n=51)	ITEC-CHF (n=91)		Control (n=93)
Age (years), mean (SD)		69.8 (13.4)	69.6 (11.7)	69.2 (11.5)		71.8 (13.0)	69.5 (12.3)		70.8 (12.4)
**Gender, n (%)**									
	Male	28 (67)	36 (86)	38 (78)		39 (77)	66 (73)		75 (81)
Weight (kg), mean (SD)		87.7 (24.1)	86.9 (19.1)	88.6 (18.3)		83.3 (19.4)	88.2 (21.0)		84.9 (19.3)
BMI (kg/m^2^), mean (SD)		31.2 (8.9)	30.2 (9.5)	31.6 (10.2)		29.1 (6.6)	31.4 (9.6)		29.6 (8.0)
NYHA^c^ class, mean (SD)		1.9 (0.6)	2.0 (0.6)	2.1 (0.4)		2.3 (0.5)	2.0 (0.5)		2.2 (0.6)
LVEF^d^ (%), mean (SD)		29.4 (6.5)	25.5 (23.7)	29.0 (7.5)		28.6 (7.9)	29.1 (7.1)		27.4 (15.9)
**Chronic condition, n (%)**									
	Type 1 diabetes	1 (2)	3 (7)	0 (0)		1 (2)	1 (1)		4 (4)
	Type 2 diabetes	9 (21)	19 (45)	19 (39)		16 (31)	28 (31)		35 (38)
	COPD^e^ or asthma	11 (26)	5 (12)	12 (25)		15 (29)	23 (25)		20 (22)
	Chronic renal disease	4 (10)	10 (24)	6 (12)		10 (20)	10 (11)		20 (22)
**Medical treatment, n (%)**									
	ACEI^f^	24 (57)	24 (57)	45 (92)		49 (96)	69 (76)		73 (79)
	Beta-blockers	30 (71)	38 (91)	46 (94)		47 (92)	76 (84)		85 (91)
	Digoxin	5 (12)	6 (14)	10 (20)		8 (16)	15 (17)		14 (15)
	Loop diuretic	28 (67)	26 (62)	42 (86)		48 (94)	70 (77)		74 (80)
	Aldosterone receptor antagonist	18 (43)	17 (41)	32 (65)		39 (77)	50 (55)		56 (60)

^a^There were no statistical differences between the characteristics of ITEC-CHF and control.

^b^ITEC-CHF: innovative telemonitoring enhanced care program for chronic heart failure.

^c^NYHA: New York Heart Association Functional Classification.

^d^LVEF: left ventricular ejection fraction.

^e^COPD: chronic obstructive pulmonary disease.

^f^ACEI: angiotensin-converting enzyme inhibitor.

### Skipped Monitoring Days in the Innovative Telemonitoring Enhanced Care Program for Chronic Heart Failure

There were 1312 participant monitoring days when weight monitoring was *skipped*. Being away from home or traveling was the major reason for skipped monitoring, occurring on 515 participant monitoring days (39.3% of total skipped days) in 41 participants. Technical issues (Table 2) resulted in skipped monitoring on 390 participant monitoring days (29.7%) in 27 participants. Hospitalizations and ED presentations caused skipped monitoring on 232 participant monitoring days (17.7%) in 18 participants, whereas health conditions such as being unwell, falling, surgery, and chemotherapy led to 136 skipped participant monitoring days (10.4%) in 11 participants. The skipped days were regarded as noncompliant with daily weighing in the analysis of the primary outcome.

**Table 2:** The percentage of technical issues in the innovative telemonitoring enhanced care program for chronic heart failure group.

Technical issues	Value, n (%)
Bluetooth connectivity	203 (52.1)
Network connectivity	61 (15.6)
Weighing scale connectivity	49 (12.6)
CHF^a^ app	34 (8.8)
Call center system/support	23 (5.9)
Weighing scale battery	18 (4.6)
Tablet battery	2 (0.4)

^a^CHF: chronic heart failure.

### Primary Outcome and Related Analysis Results

Applying the intention-to-treat analysis to the primary outcome of weight monitoring at least four days a week on average over the duration of the trial, the proportion of compliant participants in the ITEC-CHF group did not achieve statistical significance compared with that of the control group (ITEC-CHF: 67/91, 74% vs control: 56/93, 60%; *P*=.06). However, ITEC-CHF was associated with significantly more participants who monitored their body weight on average for at least 6 days per week over the duration of the trial than the control (ITEC-CHF: 41/91, 45% vs control: 23/93, 25%; *P*≤.005; Table 3).

**Table 3:** Comparison of participant compliance with daily weight monitoring between the innovative telemonitoring enhanced care program for chronic heart failure (ITEC-CHF) and the control groups. Under the conventional weight monitoring standard of at least 4 days per week, there was no significant difference between the ITEC-CHF and the control groups (*P*=.06). Under a stricter weight monitoring criterion of at least 6 days per week, more participants in the ITEC-CHF group were found to achieve this criterion than those in the control group (*P*=.005).

Compliance^a^	ITEC-CHF^b^, n (%)	Usual care, n (%)	*P* value
Participants who monitored body weight at least 6 days per week	41 (45)	23 (25)	.005
Participants who monitored body weight at least 4 days per week	67 (74)	56 (60)	.06

^a^Compliance with daily weight monitoring.

^b^ITEC-CHF: innovative telemonitoring enhanced care program for chronic heart failure.

### Secondary Outcomes

Applying a *per-protocol* analysis by excluding participants who discontinued the study (ITEC-CHF: 24/91 and control: 12/93), the difference in weight monitoring compliance was significant for weight monitoring at least 4 days a week (ITEC-CHF: 65/67, 97% vs control: 56/81, 69%; *P*<.01) and at least 6 days a week (ITEC-CHF: 41/67, 61% vs control: 23/81, 28%; *P*<.01).

In the complete case analysis, 147 participants (ITEC-CHF: 66 and control: 81) completed the Heart Failure Compliance Questionnaire at baseline and 6-month assessments (Table 4). ITEC-CHF was associated with a significantly improved score in the domains of health maintenance (*P*<.01), medication adherence (*P*<.01), and diet (*P*<.01). No significant differences were found in the category of exercise (*P*=.10), smoking (*P*=.48), or alcohol use (*P*=.32).

In the EQ-5D assessment, no significant differences were found in the change in category of mobility (*P*=.44), self-care (*P*=.26), usual activities (*P*=.59), discomfort (*P*=.46), and anxiety or depression (*P*=.38). The mean change in the overall score of EQ-5D was also not significantly different (ITEC-CHF: 4.05, SD 15.95 vs control: 1.10, SD 14.24; *P*=.13).

No significant effects were found for the 6-min walk test distance, frailty, and depression.

No significant differences were found in all-cause hospitalizations (ITEC-CHF: 73 vs control: 58; hazard ratio [HR] 1.18; *P*=.49) or emergency department (ED) presentations (ITEC-CHF: 36 vs control: 45; HR 0.83; *P*=.55), chronic heart failure (CHF)–related hospitalizations (ITEC-CHF: 15 vs control: 8; HR 1.98; *P*=.24), CHF-related ED presentations (ITEC-CHF: 4 vs control: 5; HR 0.98; *P*=.98), or unplanned hospitalizations (ITEC-CHF: 41 vs control: 39; HR 1.06; *P*=.86).

**Table 4:** Secondary outcomes of self-management behaviors, quality of life, 6-min walk test, frailty, and depression.

Compliance^a^		Baseline								6 months									Difference from baseline									*P* value
		ITEC-CHF^b^				Usual care				ITEC-CHF				Usual care				ITEC-CHF				Usual care						
		n (%)		Mean (SD)		n (%)		Mean (SD)		n (%)		Mean (SD)		n (%)		Mean (SD)		n (%)		Mean (SD)		n (%)		Mean (SD)				
Health maintenance score		90 (99)		3.83 (0.48)		93 (100)		3.77 (0.61)		67 (74)		3.88 (0.33)		81 (87)		3.60 (0.96)		66 (73)		0.06 (0.49)		81 (87)		−0.20 (1.03)		.04		
Medications score		90 (99)		3.68 (0.79)		93 (100)		3.73 (0.81)		67 (74)		3.70 (0.78)		81 (87)		3.69 (0.74)		66 (73)		0.08 (0.54)		81 (87)		−0.04 (0.87)		.05		
Diet score		90 (99)		2.56 (1.11)		93 (100)		2.81 (1.10)		67 (74)		2.97 (1.02)		81 (87)		2.81 (1.05)		66 (73)		0.35 (1.05)		81 (87)		−0.01 (1.07)		.008		
Exercise score		90 (99)		1.83 (1.19)		93 (100)		2.04 (1.30)		67 (74)		1.85 (1.33)		81 (87)		1.83 (1.34)		66 (73)		−0.03 (1.02)		81 (87)		−0.21 (1.06)		.1		
Smoking score		90 (99)		0.53 (1.43)		93 (100)		0.45 (1.26)		67 (74)		0.39 (1.18)		81 (87)		0.37 (1.13)		66 (73)		−0.14 (1.51)		81 (87)		−0.11 (1.14)		.48		
Alcohol use score		90 (99)		0.57 (1.27)		93 (100)		0.70 (1.47)		67 (74)		0.45 (1.07)		81 (87)		0.68 (1.44)		66 (73)		−0.06 (1.19)		81 (87)		0.02 (1.15)		.32		
**EQ-5D^c^**																												
	Mobility score		90 (99)		1.63 (0.84)		93 (100)		1.86 (0.90)		66 (73)		1.71 (0.95)		81 (87)		1.98 (1.03)		65 (71)		0.12 (0.72)		81 (87)		0.11 (0.81)		.44	
	Self-care score		90 (99)		1.21 (0.53)		93 (100)		1.33 (0.66)		66 (73)		1.22 (0.54)		81 (87)		1.30 (0.64)		65 (71)		0.03 (0.39)		81 (87)		−0.04 (0.62)		.26	
	Usual activities score		90 (99)		1.74 (0.94)		93 (100)		1.74 (1.01)		66 (73)		1.65 (1.01)		81 (87)		1.68 (0.92)		66 (73)		−0.06 (0.73)		81 (87)		−0.07 (0.79)		.59	
	Discomfort score		90 (99)		1.86 (0.94)		93 (100)		2.00 (0.98)		66 (73)		1.86 (1.01)		81 (87)		2.02 (0.97)		65 (71)		0.00 (0.77)		81 (87)		0.02 (0.85)		.46	
	Anxiety/depression score		90 (99)		1.87 (0.91)		93 (100)		1.82 (0.91)		66 (73)		1.85 (0.92)		81 (87)		1.79 (0.94)		65 (71)		−0.05 (0.74)		81 (87)		−0.01 (0.68)		.38	
	Your health today score		90 (99)		70.22 (18.54)		93 (100)		68.74 (17.95)		66 (73)		75.97 (20.95)		81 (87)		70.02 (18.62)		65 (71)		4.05 (15.95)		81 (87)		1.10 (14.28)		.13	
**6-min walk test**																												
	Walked distance (m)		89 (98)		367.24 (122.93)		93 (100)		350.45 (108.96)		62 (68)		396.77 (131.68)		77 (87)		367.19 (130.88)		61 (67)		17.16 (54.23)		77 (83)		10.23 (70.04)		.4	
**Frailty**																												
	Frailty score		90 (99)		3.07 (1.01)		93 (100)		3.42 (1.13)		63 (69)		2.89 (1.29)		81 (87)		3.22 (1.31)		64 (70)		−0.19 (0.94)		81 (87)		−0.23 (0.90)		.54	
**Depression**																												
	Sleep score		89 (98)		3.30 (2.10)		91 (98)		3.74 (1.93)		64 (70)		3.40 (2.04)		81 (87)		3.80 (2.00)		65 (71)		0.12 (1.59)		80 (86)		0.01 (1.72)		.5	
	Spirits score		89 (98)		2.26 (1.39)		91 (98)		2.48 (1.58)		64 (70)		2.28 (1.63)		81 (87)		2.53 (1.54)		65 (71)		0.18 (1.27)		80 (86)		0.02 (1.10)		.48	
	Tearful score		89 (98)		2.12 (1.80)		91 (98)		2.36 (1.89)		64 (70)		2.08 (1.77)		81 (87)		2.47 (1.84)		66 (73)		−0.03 (1.30)		80 (86)		0.09 (1.22)		.3	
	Frustrated score		89 (98)		3.04 (2.02)		91 (98)		3.23 (1.78)		64 (70)		2.89 (1.95)		81 (87)		2.89 (1.76)		66 (73)		−0.03 (1.48)		80 (86)		−0.21 (1.26)		.42	
	Pleasure score		89 (98)		2.39 (1.42)		91 (98)		2.59 (1.59)		64 (70)		2.23 (1.60)		81 (87)		2.46 (1.57)		66 (73)		−0.02 (1.33)		80 (86)		−0.06 (1.04)		.4	

^a^Compliance with self-management behaviors was assessed using the Heart Failure Compliance Questionnaire.

^b^The innovative telemonitoring enhanced care program for chronic heart failure (ITEC-CHF) was associated with significant improvements in the subcategories of health maintenance, medication adherence, and diet in the compliance assessment.

^c^EQ-5D: five-dimension EuroQol.

## Discussion

### Principal Findings

In this study of an innovative telemonitoring program (ITEC-CHF), facilitated by community nurses with call center support, we observed no significant differences in the weight monitoring frequency of at least 4 days a week but observed a significantly higher proportion of the intervention group achieving a weight monitoring frequency of at least 6 days per week compared with the control group receiving usual care. To our knowledge, this is the first study to use objective measures of weight monitoring and the intention-to-treat principle to comprehensively evaluate patient compliance with daily weighing in patients with CHF.

The higher weight monitoring frequency of at least 6 days per week reflects better compliance with the recommendation in contemporary clinical guidelines for patients with CHF to weigh themselves daily as a self-management strategy to maintain fluid balance and identify signs of edema [2,3]. This criterion for compliance is stricter than that applied to weight monitoring in previous studies of *most of the time* (or at least 4 days per week) and was limited by a questionnaire-based assessment of compliance, which is prone to bias [4,12].

In the intention-to-treat analysis, 45% of participants randomized to ITEC-CHF achieved a monitoring frequency of at least 6 days per week over the 6-month follow-up period of the trial. This figure was influenced by the relatively high proportion of participants who discontinued the trial from the intervention group and provides valuable insight into factors that are pertinent to telemonitoring in clinical practice. In the early stages of the trial, there were relatively frequent technical issues with the telemonitoring system, which may have led to the withdrawal of some participants. It has previously been reported that learning how to use telemonitoring technology is perceived as burdensome and creates anxiety in some patients, especially those who are older [24]. This may be further exacerbated in the event of technical issues. Technical issues also resulted in increased reliance on technical support, which would have increased the cost of telemonitoring, although this was not assessed in this study, highlighting the need for future telemonitoring studies with a health economics component. These issues highlight the importance of telemonitoring systems being seamless and reliable to not create an unnecessary burden on patients and their carers or health service providers [25]. Therefore, they underscore the need to improve the reliability and user experience of telemonitoring systems [26] for use in clinical CHF care.

In participants who completed the trial (the cohort in which the per-protocol analysis was conducted), compliance with weight monitoring in ITEC-CHF was high; 97% of participants monitored themselves for at least 4 days a week over the 6-month duration of the trial, and 61% of participants monitored themselves at least 6 days a week. In the ITEC-CHF group, 390 participant monitoring days were *skipped* because of technical issues, meaning that the difference in weight monitoring compliance between the ITEC-CHF and control groups is likely to be underestimated. These positive findings regarding weight monitoring compliance in participants who adhered to the program are likely to be underpinned by the multifactorial support mechanisms provided. This is further supported by a substantial number of alerts reported in this study, which included the automated generation of reminder alerts on 715 patient days when a weight recording was not received by 10 AM as well as contact made by the call center and project nurses. These findings not only demonstrated the effectiveness of ITEC-CHF in supporting weight management but also indicated a strong need for such support in ongoing CHF care.

The ITEC-CHF group experienced a significant improvement in health maintenance compared with usual care, as measured by the Heart Failure Compliance Questionnaire. This positive result was consistent with a finding of improved self-care maintenance reported in 2 other RCTs of telemonitoring in CHF [27,28]. Similarly, there was a significant improvement in adherence to medication and diet recommendations in the ITEC-CHF group, but not in the control group. These findings imply increased engagement with the heart failure nurses that occurred following the alerts generated through the telemonitoring intervention. These interactions created the opportunity for *teachable moments*, enabling nurses to provide informal education to reinforce self-management practices. It has previously been acknowledged that patients often benefit from ongoing support in CHF care to effectively manage their health conditions through reinforcement of self-management strategies [29]. It is also possible that merely being monitored was sufficient to enhance adherence to more desirable patterns of care because of a surveillance effect [30]. Nevertheless, the combination of telemonitoring with nursing support resulted in improved self-management activities, although it is often difficult for researchers to identify which component of the ITEC-CHF program drove these improvements.

There were no significant effects of ITEC-CHF on hospitalizations and ED presentations, although it should be noted that the study was underpowered for this analysis. We observed that CHF-related events were not the major cause of hospitalizations (23/131, 17.5%) or ED presentations (9/81, 11%). CHF is a complex condition, more prevalent in older people, and associated with a range of comorbidities. Telemonitoring in the context of this study focused exclusively on daily weight recordings. This finding implies a need to extend telemonitoring intervention for comorbidity and critical non-CHF–related health conditions to more comprehensively address the range of health issues faced by patients with CHF. To date, the effectiveness of telemonitoring in improving hospitalization and mortality remains inconclusive [13,14]. However, it has been shown that reduction in health services utilization, including unscheduled hospitalizations and length of stay, can be achieved for broader chronic disease management by monitoring a range of vital signs using telemonitoring enhanced care coordination [31,32]. Further research to understand the underlying principles that impact hospitalizations and ED presentations related to a specific primary diagnosis such as CHF remains essential in future studies.

There are several limitations of this study that warrant discussion. First, the study was limited to a 6-month intervention, which may have been insufficient to translate to meaningful changes in clinical characteristics of patients. In addition, a relatively high number of participants discontinued their involvement in the trial, which has the potential to bias the analysis of some secondary outcomes where the intention-to-treat principle could not be applied. High rates of discontinuation in the ITEC-CHF group suggest a potential bias in the per-protocol analysis. Participants who discontinued in the ITEC-CHF group were unlikely to be random because some were because of deteriorating health (palliative care or dialysis) and because some were deceased, as shown in Figure 1. Therefore, we did not use the multiple imputation approach outlined in the trial protocol [15]. The study did not achieve the target sample size (n=300), proposed in the trial protocol [15]; this might have compromised the power to detect significant effects in the analysis of the primary outcome. There are several reasons for the smaller than proposed sample size. We experienced technical issues with the telemonitoring software that were not apparent during an extensive testing phase before study commencement, which delayed recruitment. This highlights the challenges that arise in a real-world environment that may not be present in a testing scenario. We also experienced slower than anticipated recruitment; some patients were reluctant to engage in a model of care involving technology, whereas others were concerned about their ability to weigh themselves safely because of frailty and were therefore excluded from the trial. A substantial number of patients reported as having CHF did not have an echocardiogram documented in their medical records and were therefore excluded because they failed to meet the inclusion criteria of an EF of <40%. Finally, a high proportion of patients who were residents of nursing homes were excluded on this basis. These issues highlight that the clinical complexity of many patients with CHF, who are often older with multiple comorbidities, may complicate their ability to engage with telemonitoring and underpins the importance of telemonitoring models being developed that are reliable, easy to use, and accessible for patients across the clinical spectrum of CHF. Finally, the patient compliance rate in usual care (25% for at least 6 days per week and 60% for at least 4 days per week) was likely to be influenced by the provision of weight scales to participants, which kept a record of their weight recordings and, accordingly, might have resulted in a Hawthorne effect [33], reducing the difference between the ITEC-CHF and control groups.

### Conclusions

The ITEC-CHF study is the first to report the effects of telemonitoring on weight monitoring compliance using an objective measure of weight recordings in patients with CHF. The proportion of participants in the ITEC-CHF program achieving a weight monitoring frequency of at least 6 days per week was higher than that in usual care controls. Furthermore, ITEC-CHF resulted in significant improvements in CHF self-management related to health maintenance, medication adherence, and diet. Among participants who completed the study, there was a high level of compliance with weight monitoring, underscoring the importance of telemonitoring platforms that are seamless to reduce the risk of patients disengaging with the technology. Although telemonitoring and digital health more broadly offer significant potential for supporting patients in self-managing chronic conditions such as heart failure, further research is required to refine these evolving strategies to achieve effective care outcomes.

## Appendix

Multimedia Appendix 1 User interface.

Multimedia Appendix 2 Bluetooth-enabled scales.

Multimedia Appendix 3 International Classification of Diseases, Tenth Revision, Clinical Modification, diagnosis codes used to determine heart failure–related hospitalizations and emergency department presentations.

Multimedia Appendix 4 CONSORT-eHEALTH checklist (V 1.6.1).

## Abbreviations

CHF: chronic heart failure
ED: emergency department
EF: ejection fraction
EQ-5D: five-dimension EuroQol
GP: general practitioner
ITEC-CHF: innovative telemonitoring enhanced care program for chronic heart failure
MMH: Manage My Health
RCT: randomized controlled trial
