# Supplementary figures and images for: The Effects of Telemonitoring on Patient Compliance With Self-Management Recommendations and Outcomes of the Innovative Telemonitoring Enhanced Care Program for Chronic Heart Failure: Randomized Controlled Trial
Source: J Med Internet Res. 2020 Jul 8;22(7):e17559. doi: 10.2196/17559 (PMC7381046; doi:10.2196/17559)

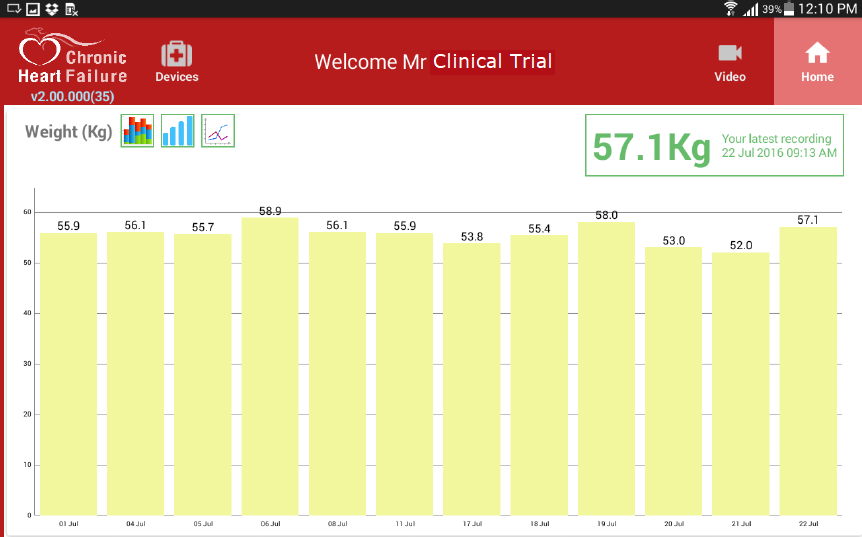

Supplement: Multimedia Appendix 1 [file jmir_v22i7e17559_app1.PNG]

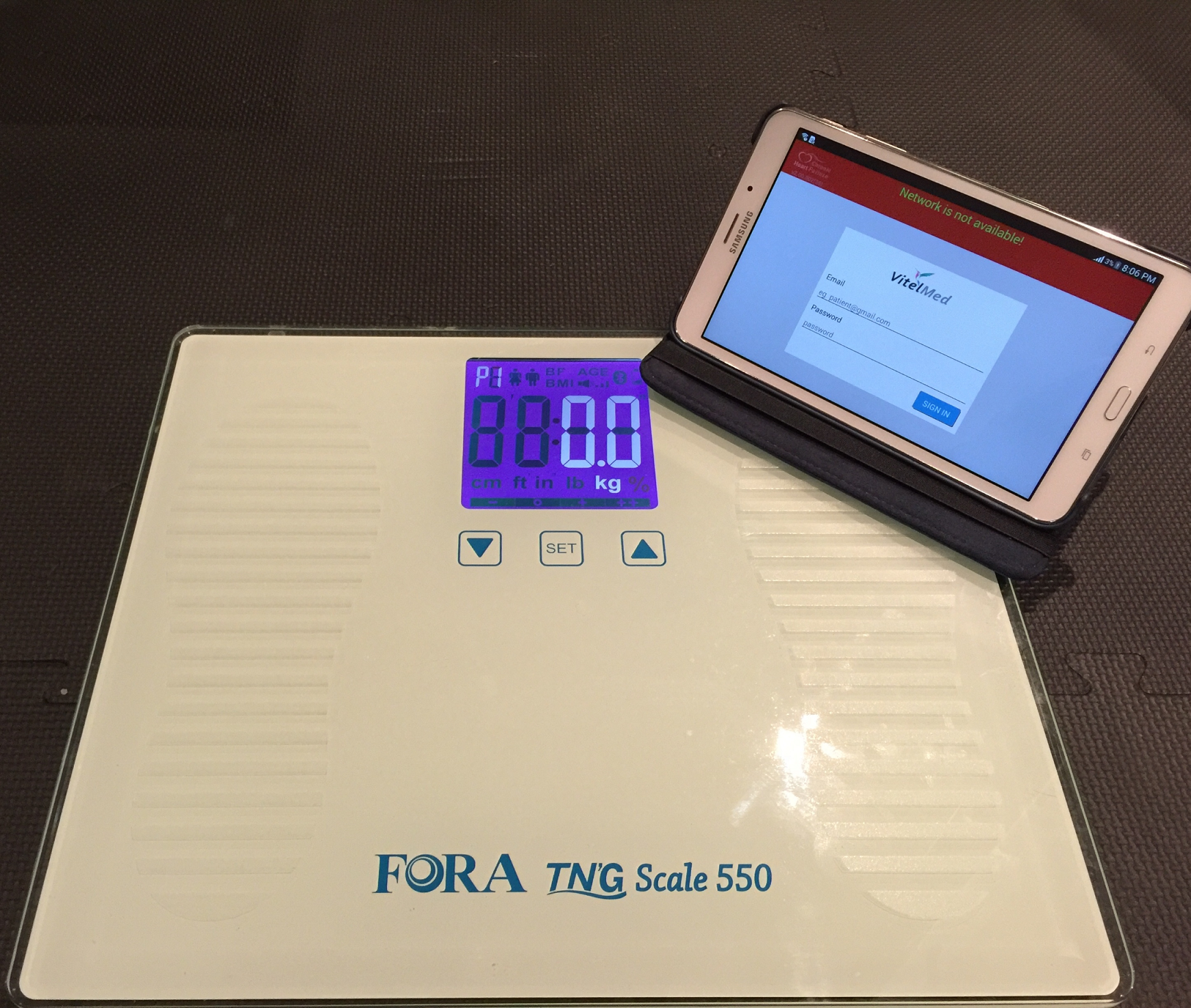

Supplement: Multimedia Appendix 2 [file jmir_v22i7e17559_app2.png]
