# Supplementary material for: The Effects of Telemonitoring on Patient Compliance With Self-Management Recommendations and Outcomes of the Innovative Telemonitoring Enhanced Care Program for Chronic Heart Failure: Randomized Controlled Trial
Source: J Med Internet Res. 2020 Jul 8;22(7):e17559. doi: 10.2196/17559 (PMC7381046; doi:10.2196/17559)
Supplement: Multimedia Appendix 3 [file jmir_v22i7e17559_app3.docx]

| **ICD-10-CM** | **Description** |
| --- | --- |
| I25.5 | Ischaemic cardiomyopathy. |
| I42 | Cardiomyopathy. |
| I42.0 | Dilated cardiomyopathy. |
| I42.6 | Alcoholic cardiomyopathy. |
| I42.7 | Cardiomyopathy due to drugs and other external agents. |
| I42.8 | Other cardiomyopathies |
| I42.9 | Cardiomyopathy, unspecified. |
| I50 | Heart failure. |
| I50.1 | Left ventricular failure, unspecified. |
| I50.2 | Systolic (congestive) heart failure. |
| I50.4 | Combined systolic (congestive) and diastolic (congestive) heart failure. |
| I50.9 | Heart failure, unspecified. |
| I50.20 | Unspecified systolic (congestive) heart failure. |
| I50.21 | Acute systolic (congestive) heart failure. |
| I50.22 | Chronic systolic (congestive) heart failure. |
| I50.23 | Acute on chronic systolic (congestive) heart failure. |
| I50.40 | Unspecified combined systolic (congestive) and diastolic (congestive) heart failure. |
| I50.41 | Acute combined systolic (congestive) and diastolic (congestive) heart failure. |
| I50.42 | Chronic combined systolic (congestive) and diastolic (congestive) heart failure. |
| I50.43 | Acute on chronic combined systolic (congestive) and diastolic (congestive) heart failure. |
